# High-quality genome assembly of a *C. crossoptilon* and related functional and genetics data resources

**DOI:** 10.1038/s41597-024-03087-5

**Published:** 2024-02-27

**Authors:** Siwen Wu, Kun Wang, Tengfei Dou, Sisi Yuan, Dong-Dong Wu, Changrong Ge, Junjing Jia, Zhengchang Su

**Affiliations:** 1 Department of Bioinformatics and Genomics, College of Computing and Informatics, the University of North Carolina at Charlotte, Charlotte, NC 28223 USA; 2 Faculty of Animal Science and Technology, Yunnan Agricultural University, Kunming, 650201 Yunnan China; 3 State Key Laboratory of Genetic Resources and Evolution/Key Laboratory of Healthy Aging Research of Yunnan Province, Kunming Institute of Zoology, Chinese Academy of Sciences, Kunming, Yunnan China

**Keywords:** Genome, Genome assembly algorithms

## Abstract

There are four species in the *Crossoptilon* genus inhibiting at from very low to very high altitudes across China, and they are in varying levels of danger of extinction. To better understand the genetic basis of adaptation to high altitudes and genetic changes due to bottleneck, we assembled the genome (~1.02 Gb) of a white eared pheasant (WT) (*Crossoptilon crossoptilon*) inhibiting at high altitudes (3,000~7,000 m) in northwest of Yunnan province, China, using a combination of Illumina short reads, PacBio long reads and Hi-C reads, with a contig N50 of 19.63 Mb and only six gaps. To further provide resources for gene annotation as well as functional and population genetics analyses, we sequenced transcriptomes of 20 major tissues of the WT individual and re-sequenced another 10 WT individuals and a blue eared pheasant (*Crossoptilon auritum*) individual inhabiting at intermediate altitudes (1,500~3,000 m). Our assembled WT genome, transcriptome data, and DNA sequencing data can be valuable resources for studying the biology, evolution and developing conservation strategies of these endangered species.

## Background & Summary

*Crossoptilon*, belonging to the Phasianidae family in the Galliformes order, is a rare but important genus endemic in China^1^. There are four species in the *Crossoptilon* genus, including Tibetan eared pheasant (TB) (C. *harmani*), white eared pheasant (WT) (C. *crossoptilon*), blue eared pheasant (BL) (C. *auritum*) and brown eared pheasant (BR) (C. *mantchuricum*)^2,3^. TBs are only found in southeastern Tibet with high altitudes (more than 6,000 m), BRs are mainly distributed in mountains of Beijing, Shanxi and Hebei provinces with low altitudes (20~1,000 m)^2^, BLs are only encountered in the mountains of Qinghai, Gansu and Sichuan provinces and Ningxia Autonomous Region with intermediate altitudes (1,500~3,000 m)^2^, and WTs are distributed in Qinghai, Sichuan, Yunnan and Tibet Province of China with high altitudes (3,000~7,000 m)^2^. All the four species are of high commercial value but in varying levels of danger of extinction, and thus are national key protection animals in China. They are also excellent model organisms for studying genetics basis of altitude adaptation of closely related species and genetic changes during the bottleneck of endangered species. However, studies of the four species are rare, and mostly limited to single genes, partial sequences^4,5^ or mitochondrial DNA sequences^1,6^. Although the genome of a BR individual was sequenced and assembled in 2020^7^ using Illumina short reads and fragment libraries, with a contig N50 of 0.11 Mb, a scaffold N50 of 3.63 Mb and a BUSCO complete value^8^ of 95.1%, it is not continuous and accurate enough for various genomic studies of the *Crossoptilon* species. Therefore, it is urgent to sequence and assemble a *Crossoptilon* species genome with high-quality. Moreover, although re-sequencing data of varying numbers of BR and BL individuals have been available^7^, no WT and TB individuals have been so far sequenced, hampering population genetics studies of the *Crossoptilon* species.

To fill these gapes at least partially, we assembled the genome of a WT female individual at the chromosome-level with very high quality using a combination of Illumina short reads, PacBio long reads and Hi-C reads. The resulting assembly has a total length of 1.02 Gb, with a contig N50 of 19.6 Mb, a scaffold N50 of 29.6 Mb, a complete BUSCO value^8^ of 97.2% and only six gaps. To facilitate the annotation and functional analysis of the genome, we also sequenced transcriptomes of 20 major tissues of the WT individual. Moreover, we re-sequenced another 10 WT individuals and one individual of BL for population genetics and comparative genomics analyses. Therefore, the assembled almost-gap-free WT genome as well as the large volumes of transcriptome and DNA sequencing data can be valuable resources for studying the biology, evolution and developing conservation strategies of these endangered valuable species.

## Methods

### Sample information

Blood samples of a total of 10 WT individuals (five males and six females) aged about 10 months were collected from Diqing Tibet Autonomous prefecture, Yannan Province, China and subjected to Illumina paired-end DNA short reads sequencing. A female individual was collected from the same area for whole genome assembly, and its relevant tissues were subject to Illumina paired-end DNA short reads sequencing, PacBio long reads sequencing and Hi-C paired-end short reads sequencing. Moreover, 20 tissues (Heart, Liver, Spleen, Lung, Kidney, Pancreas, Gizzard, Glandular, Crops, Ovary, Abdominal fat, Rectum, Duodenum, Cecum, Skin, Small intestine, Brain, Cerebellum, Chest muscle, Leg muscle) of the WT individual were subject to paired-end RNA-seq. Furthermore, blood sample of a BL individual (female) was collected from Guangzhou Zoo, China and subjected to Illumina paired-end DNA short reads sequencing.

### Ethics approval

All the experimental procedures were approved by the Animal Care and Use Committee of the Yunnan Agricultural University (approval ID: YAU202103047). The care and use of animals fully complied with local animal welfare laws, guidelines, and policies.

### Short reads DNA sequencing

Two milliliters of blood were drawn from the wing vein of each bird in a centrifuge tube containing anticoagulant (EDTA-2K) and stored at −80 °C until use. Genomic DNA (10 µg) in each blood sample was extracted using a DNA extraction kit (DP326, TIANGEN Biotech, Beijing, China) and fragmented using a Bioruptor Pico System (Diagenode, Belgium). DNA fragments around 350 bp were selected using SPRI beads (Beckman Coulter, IN, USA). DNA-sequencing libraries were prepared using Illumina TruSeq® DNA Library Prep Kits (Illumina, CA, USA) following the vendor’s instructions. The libraries were subject to 150 cycles paired-end sequencing on an Illumina Novaseq. 6000 platform (Illumina, CA, USA) at 102X coverage.

### PacBio long reads DNA sequencing

High molecular weight DNA was extracted from the blood sample using NANOBIND® DNA Extraction Kits (PacBio, CA, USA) following the vendor’s instructions. DNA fragments of about 25 kb were size-selected using a BluePippin system (Sage Science, MA, USA). Sequencing libraries were prepared for the DNA fragments using SMRTbell® prep kits (PacBio, CA, USA) following the vendor’s instructions, and subsequently sequenced on a PacBio Sequel II platform (PacBio, CA, USA) at 91X coverage.

### Transcriptome sequencing

One to two grams of tissues (Heart, Liver, Spleen, Lung, Kidney, Pancreas, Gizzard, Glandular, Crops, Ovary, Abdominal fat, Rectum, Duodenum, Cecum, Skin, Small intestine, Brain, Cerebellum, Chest muscle, Leg muscle) were collected from the selected female WT individual in a centrifuge tube and immediately frozen in liquid nitrogen, then stored at −80 °C until use. Total RNA from each tissue sample were extracted from each tissue or mixed tissues using TRlzol reagents (TIANGEN Biotech, Beijing China) according to the manufacturer’s instructions. RNA-sequencing libraries for each tissue collected from the individual were prepared using Illumina TruSeq® RNA Library Prep Kits (Illumina, San Diego) following the vendor’s instructions. The libraries were subject to 150 cycles paired-end sequencing on an Illumina Novaseq. 6000 platform with a total of 936,231,391 pairs of reads.

### Hi-C reads sequencing

Five milliliters of blood were drawn from the wing vein of the selected WT individual in a Streck Cell-free DNA BCT collecting vessel (Streck Corporate, USA), and stored at 4 °C and used in 24 hours. Hi-C libraries were constructed using Phase Genomics’ Animal Hi-C kit following the vendor’s instructions and subsequently sequenced on an Illumina’s Novaseq. 6000 platform at a sequencing depth of 81X.

### Cleaning of raw sequencing reads

For the short sequencing reads, we removed possible adaptors and low-quality portions using TrimGalore (https://github.com/FelixKrueger/TrimGalore) with parameters length > 50 and q > 10. For the long sequencing reads, we removed the reads shorter than 5,000 bp.

### Quality assessment of sequencing data

We used FastQC (0.12.1) (http://www.bioinformatics.babraham.ac.uk/projects/fastqc) to evaluate the quality of different kinds of sequencing data of the WT and BL.

### Contig assembling and scaffolding

We used the PacBio long reads to assemble the contigs using Wtdbg (2.5)^9^ (parameters used: -x ccs -g 1 g -X 98 -e 6), and polished the contigs using Wtdbg (2.5)^9^ with Illumina DNA short reads for the WT (default settings). Then we used SALSA^10,11^ to bridge the contigs and obtain the scaffolds with Hi-C short reads (parameters used: -e AAGCTT -m yes -i 4 -s 1000000000 -c 500). We filled the gaps in the scaffolds using PBJelly^12^ with the PacBio long reads (parameters used: --minMatch 8 --minPctSimilarity 70 --bestn 1 --nCandidates 20 --maxScore ‐500), and then made two rounds of polish by firstly using Racon (1.4.21)^13^ with PacBio long reads (default settings) and secondly using NextPolish (1.4.0)^14^ with Illumina DNA short reads from the selected WT individual (default settings).

### Quality evaluation of the assembly

We masked the repeats for the assembly of the WT genome using WindowMasker (2.11.0)^15^ to get the repeat rate, and estimated the heterozygosity of the assembly using Jellyfish (2.3.0)^16^ and GenomeScope^17^. To estimate the continuity of the assembly, we used QUAST (5.0.2)^18^ to calculate the contig N50 and scaffold N50. To estimate the structural accuracy, we used Asset (https://github.com/dfguan/asset) to calculate the reliable block N50 and used BUSCO (5.1.3)^8^ against the aves_odb10 database to calculate the false duplication rate for the assembly. To estimate the base accuracy, we used Merqury (1.3)^19^ to calculate the *k-mer* QV (k = 17) and *k-mer* completeness for the assembly, used BWA (0.7.17)^20^ to map the short reads of the selected WT individual to the assembly, and used SAMtools (1.10)^21^ to analyze the mapping results. To estimate the functional completeness, we used BUSCO (5.1.3)^8^ to assess the completeness of the assembly against the aves_odb10 database. To plot the heatmap of the scaffolds of the assembly, we mapped the Hi-C paired-end short reads to the assembly using BWA (0.7.17)^20^, used SAMtools (1.10)^21^ and Pairtools (0.3.0) (https://github.com/open2c/pairtools) to analyze the mapping results, and used Higlass^22^ to plot the heatmap for the assembly. Default settings were used in each tool.

## Data Records

The Illumina DNA paired-end short reads, PacBio long reads, Hi-C paired-end short reads and the RNA-seq paired-end short reads of different tissues of the selected WT individual are available at NCBI SRA with the accession number PRJNA956489^23^. The re-sequencing paired-end short reads of the other 10 WT individuals are available at NCBI SRA with the accession number PRJNA956570^24^. The re-sequencing paired-end short reads of the BL individual are available at NCBI SRA with the accession number PRJNA1039024^25^. The assembled genome of the WT individual is available at GenBank with the accession number PRJNA956505^26^.

## Technical Validation

### Quality evaluation of the sequencing data

We generated Illumina DNA paired-end short reads, PacBio long reads and Hi-C paired-end short reads for a female WT individual. As shown in Table 1, for the Illumina DNA paired-end short reads, the sequencing length is 150 bp and the sequencing depth is 102X. For the PacBio long reads, the average sequencing length is 10 kbp and the sequencing depth is 91X. For the Hi-C paired-end short reads, the sequencing length is 150 bp and the sequencing depth is 81X. In addition, we also sequenced the transcriptomes of 20 tissues (Heart, Liver, Spleen, Lung, Kidney, Pancreas, Gizzard, Glandular, Crops, Ovary, Abdominal fat, Rectum, Duodenum, Cecum, Skin, Small intestine, Brain, Cerebellum, Chest muscle, Leg muscle) of the WT individual and re-sequenced another 10 individuals of WT. As shown in Table 1, for the RNA-seq reads, the sequencing length is 150 bp with a total of 936,231,391 pairs of reads. For the re-sequencing reads, the sequencing length is 150 bp and the average sequencing depth is 31X. For the re-sequencing reads of the BL individual, the sequencing length is 150 bp and the average sequencing depth is 52X.

**Table 1:** Summary of raw sequencing data.

WT Short reads	Depth	102X
	Length	150 bp
	# Pairs	33,85,15,576
WT Long reads	Depth	91X
	Average reads length	10 kbp
	# Total reads	89,82,129
	# Reads > 5 kbp	55,57,754
WT Hi-C reads	Depth	81X
	Length	150 bp
	# Pairs	26,96,97,038
WT RNA-seq reads	Length	150 bp
	# Tissues	20
	# Pairs	93,62,33,391
WT Re-sequencing	# Individuals	10
	Average depth	31X
	Length	150 bp
BL Re-sequencing	# Individuals	1
	Depth	52X
	Length	150 bp

**Fig. 1:**
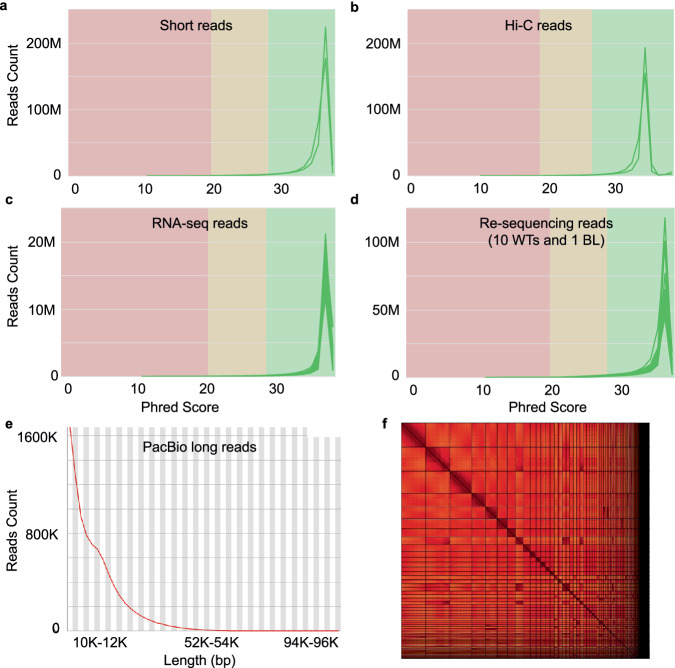
Quality assessment of different types of sequencing reads and the assembled WT genomes. (**a**) Number of short reads of the WT individual with the indicated phred scores. (**b**) Number of Hi-C short reads of the WT individual with the indicated phred scores. (**c**) Number of RNA-seq reads in each of the 20 libraries of the tissues of the WT individual with the indicated phred scores. (**d**) Number of re-sequencing short reads in each of the libraries of the 10 WT individuals and the BL individual with the indicated phred scores. Notably, two paired-end reads in each library are evaluated separately. (**e**) Number of PacBio long reads with the indicated lengths. (**f**) Hi-C interaction heatmap of the scaffolds of the WT assembly, sorted by the scaffold size.

Figure 1a–e show the quality assessment of the different sequencing data. All the Illumina DNA paired-end short reads, Hi-C paired-end reads, RNA-seq reads and re-sequencing reads (WT and BL) have a Phred score greater than 35 (Fig. 1a–d), suggesting that the base accuracy of all these reads is greater than 99.9% (https://www.illumina.com/documents/products/technotes/technote_Q-Scores.pdf) and are of very high quality. As the PacBio long reads do not come with Phred quality scores, we evaluated their quality using length distribution. As shown in Fig. 1e, the PacBio long reads have an average length about 10 kbp, indicating that they are of high quality.

### Evaluation of the quality of the assembled WT genome

Using the short and long sequencing reads, we assembled the genome of the WT individual into 805 contigs with a contig N50 of 19.63 Mb and a total contig length of 1.02 Gb, comparable to those of the chicken (*Gallus gallus*) genome assemblies GRCg6a and GRCg7b/w as well as of the previously assembled BR genome^7^ (1.01 Gb) (Table 2). Using the Hi-C paired-end short reads, we further assembled the contigs into 643 scaffolds with a scaffold N50 of 29.59 Mb (Table 2). We assessed the quality of the assembly using the criteria proposed by the VGP consortium^27^, and compared it with chicken assemblies GRCg6a and GRCg7b/w, the best-studied bird genomes. These criteria include genome features (heterozygosity and repeat rates), continuity (assembly size, N50 and gaps), structure accuracy (reliable block N50 and false duplication rate), base accuracy (k-mer QV, k-mer completeness and short reads mapping rate) and functional completeness (BUSCO completeness) (Table 2).

**Table 2:** Evaluation of the quality of the assembled WT individual genome.

Breed	Genome		Continuity				Structural Accuracy		Base Accuracy			Funtional Completeness
	Het (%)	Rep (%)	Size (Gb)	# Contigs (Contig N50) (Mb)	# Scaffolds (Scaffold N50) (Mb)	# Gaps	Reliable block N50 (Mb)	False duplications (%)	k-mer QV	k-mer Completeness (%)	Short reads Completeness (%)	BUSCO Completeness (%)
WT	0.54	20.6	1.023	805 (19.6)	643 (29.6)	6	14.6	0.3	42.0	95.3	99.3	97.2
GRCg6a	—	20.6	1.056	1,402 (17.7)	464 (20.8)	500,945	—	0.4	—	—	—	96.6
GRCg7b	—	20.6	1.050	677 (18.8)	214 (90.9)	463	—	0.4	—	—	—	96.6
GRCg7w	—	20.2	1.046	685 (17.7)	276 (90.6)	409	—	0.4	—	—	—	96.8

The heterozygosity rate of the assembled WT genome is 0.54%, and its repeat rate is 20.6%, both are comparable to those of the GRCg6a and GRCg7b/w assemblies (Table 2). For the continuity, the contig N50 (19.6 Mb) of the assembly is slightly larger than those of the GRCg6a (17.7 Mb) and GRCg7b/w (18.8/17.7 Mb) assemblies. The scaffold N50 (29.6 Mb) of the assembly is slightly larger than that of the GRCg6a assembly (20.8 Mb), but smaller than those of the GRCg7b/w assemblies (90.9/90.6 Mb) (Table 2). For gaps, there are only six gaps in our assembly, which is much fewer than those of the GRCg6a (500,945) and GRCg7b/w (463/409) assemblies (Table 2), indicating that our assembly is almost gapless. For the structural accuracy, the reliable block N50 of our assembly (14.6 Mb) is comparable to those of Avian genomes assembled by the recent VGP consortium^27^. The false duplication rate^8^ of our assembly (0.3%) is slightly smaller than those of the GRCg6a (0.4%) and GRCg7b/w (0.4%) assemblies (Table 2), indicating that the structural accuracy of our assembly is very high. For the base accuracy, the k-mer QV of our assembly is 42.0, suggesting that the consensus base accuracy is greater than 99.99%^19^ (Table 2). The k-mer completeness (defined as the fraction of reliable k-mers in highly accurate short reads data that are also found in the assembly^19^) of our assembly is 95.3% (Table 2), which is comparable to those of the recent VGP assemblies^27^. To further evaluate the base accuracy, we mapped the Illumina short reads of the WT individual to the assembly and found that 99.3% short reads can be mapped to the assembly (Table 2), suggesting that our assembly is of high base accuracy. For the functional completeness, we achieved a larger BUSCO completeness value^8^ (97.2%) than those of the GRCg6a (96.6%) and GRCg7b/w (96.6%/96.8%) assemblies (Table 2), suggesting that our assembly is of high functional completeness. To further check whether our assembly is at chromosome-level, we plotted the Hi-C interaction heatmap of the scaffolds. As shown in Fig. 1f, almost all the scaffolds form a square at the diagonal of the heatmap, indicating that our assembly is at chromosome-level, although we lack genetic marks to sort them into specific chromosomes.

## Usage Notes

Our almost gapless assembly of the WT genome can be used jointly with other assembled high-quality bird genomes to study many important questions in bird biology and evolution. The WT genome can be compared with the previously assembled BR genome^7^ to reveal their genetic basis to adapt to high and low altitude niches, respectively. The WT genome can also be used as a reference to call single nucleotide variants in the populations of the *Crossoptilon* species, thereby identifying the natural selective sweeps in their genomes. The RNA-seq data generated from the 20 tissues of the WT individual can be used to annotate its assembled genome and the previously assembled BR genome^7^. The RNA-seq data can also be used in other functional analyses of the species. The re-sequencing WT and BL data together with other available re-sequencing data from BR and BL populations^7^ can be used to identify natural selective signatures on the WT, BL and BR genomes to reveal their genetic bases to adapt to high, intermediate and low altitude niches, respectively. These data can also be used to reveal genetic changes during the bottleneck of these endangered species as previously demonstrated^7^, thereby developing conservation strategies to more effectively protect these endangered valuable species.

## Data Availability

All genome assembly code and the corresponding pipeline description are available at https://github.com/zhengchangsulab/A-genome-assebmly-and-annotation-pipeline.
